# Scaling Up Patient-Centered Psychological Treatments for Perinatal Depression in the Wake of a Global Pandemic

**DOI:** 10.3389/fpsyt.2021.826019

**Published:** 2022-02-07

**Authors:** Daisy R. Singla, Samantha Meltzer-Brody, Katarina Savel, Richard K. Silver

**Affiliations:** ^1^Campbell Family Mental Health Research Institute, Centre for Addiction and Mental Health, Toronto, ON, Canada; ^2^Lunenfeld Tanenbaum Research Institute, Sinai Health, Toronto, ON, Canada; ^3^Department of Psychiatry, Temerty Faculty of Medicine, University of Toronto, Toronto, ON, Canada; ^4^Department of Psychiatry, University of North Carolina at Chapel Hill, Chapel Hill, NC, United States; ^5^Department of Obstetrics and Gynecology, North Shore University Health System, University of Chicago, Chicago, IL, United States

**Keywords:** task sharing, perinatal depression, health equity, telemedicine, psychotherapy

## Abstract

There is a call to action to reduce the public health burden of perinatal depression worldwide. The COVID-19 pandemic has further highlighted significant gaps in perinatal mental health care, especially among women who identify as Black, Indigenous, People of Color (BIPOC). While psychotherapeutic (cognitive, behavioral and interpersonal) interventions are endorsed for perinatal mood disorders, barriers to access and uptake contribute to inequitable access to treatment at the population level. To effectively address these barriers and increase the scalability of psychotherapy among perinatal women, we suggest four pragmatic questions to be answered from a patient-centered lens; namely, “who,” “what,” “how,” and “when.” Promising avenues include task-sharing among mental health non-specialists, an emphasis on culturally sensitive care, web-based delivery of psychotherapy with some caveats, and a lifespan approach to perinatal mental health. Innovative research efforts are seeking to validate these approaches in diverse contexts across North America and the UK, lending optimism toward scalable and long-term solutions for equitable perinatal mental health care.

## Introduction

The past 2 years have highlighted that barriers and mental health problems are exacerbated for perinatal populations during COVID (1, 2), and even more so for women of color (3, 4). Perinatal (pregnant and postpartum) women face major psychological stressors that put them at higher risk of developing common mental health disorders, such as depression and anxiety (5). Yet limited access to and uptake of traditional mental healthcare has resulted in inequitable access to treatment, especially given the COVID-19 pandemic.

Cognitive, behavioral and interpersonal therapies are all effective in addressing perinatal depression and comorbid anxiety. The US Preventive Services Task Force (UTPSTF) made headlines in 2019 by endorsing evidence-based psychotherapy as a preventative strategy for women at-risk for perinatal mood disorders (6). While the UTPSTF endorsement of psychotherapies for perinatal mood disorders was a welcome public health development, adoption of this recommendation poses significant challenges to our current models for delivering these interventions at the population level. In the USA and Canada, as few as 20% of women have access to minimally-adequate treatments for depression including psychotherapy (6), and other substantive barriers to treatment include transportation, childcare obligations, and mental health stigma. Proven treatment strategies that are available are currently compromised by a critical shortage of mental health professionals and a lack of patient-centered, culturally-sensitive care. Furthermore, there is a substantial gap in the existing literature on delivery of psychotherapies, including a dearth of large-scale studies, limited evaluation of the implementation of proven technological innovations and a lack of individually-tailored intervention trials (7).

Overcoming this dilemma requires innovative solutions to address four salient questions; namely, “who,” “what,” “how,” and “when” evidence-based psychotherapies can be delivered from a patient-centered lens. Answers to these questions will go a long way toward a solution to this pressing public health issue that has been made worse by the pandemic.

### Who

There will never be enough specialist providers to address the treatment gap for perinatal depression and anxiety. One innovation to improve access to care is task sharing—the rational redistribution of tasks (8) to train non (mental health) specialist providers (NSPs) to deliver psychotherapy with appropriate levels of supervision. NSPs are individuals with no specialized degree in mental health and range from community health workers, peers and lay counselors, to midwives, teachers, and nurses. The specific type of NSP depends on the context. In the past three decades, task sharing has gained a growing popularity worldwide with applications for perinatal mental health specifically in low- and middle-income countries (9), as well as high-income countries, such as the USA and Canada (10). NSP-delivered mental health interventions were shown to be effective across the globe, targeting primary care attendees, with a range of depressive symptoms, including those at high-risk based on self-report measures. Notably, these populations were ethnically diverse, including low-income and minority groups as well as urban populations. Further research is required to test models including other key family members, including partners and children. Both have been successfully integrated within NSP-delivered interventions in low-resource settings in India (11), Pakistan (12), and Uganda (13).

A key question is overcoming professional guilds to best address how to implement this model within pragmatic contexts and whether they are sustainable (14). Evidence is also lacking as to whether specialists and non-specialists are comparable in resolving perinatal depressive symptoms when delivering *identical* treatment. The opportunity to train and leverage a cadre of capable paraprofessionals, some of whom are already embedded in the existing obstetrical care team, has significant implications for access, feasibility and cost of treatment.

### What

While psychotherapies are generally effective across cultural groups and problem areas (15), engagement remains significantly lower among BIPOC (Black, Indigenous, Persons of Color) perinatal women compared to their white counterparts (16). BIPOC women are less likely to access and continue mental health services (17, 18) due to racialized barriers that include experiences of discrimination and racial micro-aggression, mistrust of healthcare providers, and reduced quality of services (18–20). In a recent study of 30 perinatal women from the United States, greater attention to cultural factors and social determinants of health was highly recommended (21). An explicit focus on culturally-sensitive and patient-centered care for BIPOC perinatal women is needed.

One solution that some evidence-based psychotherapies offer is to facilitate an idiosyncratic and process-oriented model. By idiosyncratic, the therapy provider collaboratively explores and often maps the patient's individual context, individual values as well as goals and behaviors to guide the content of the conversation while expressing a supportive and empathic stance (22). Furthermore, and to better understand the context in which the patient's symptoms occur, providers are encouraged to explicitly ask their patients whether background or identity make a difference to or intersect with their problem. This individualized approach is ultimately guided by a patient-centered approach that promotes partnership between patient and provider, rather than hierarchy. In addition, process oriented models emphasize the dynamics of the interaction occurring between the provider and the patient (15). These models take into account how cultural meaning is ascribed to treatment contexts rather than how culture matters for different groups (23, 24). For example, in his “shifting cultural lens” model, Lopez et al. (23) suggests that cultural competency involves the ability of the provider to move between two cultural perspectives in understanding the culturally-based meaning of clients from diverse cultural backgrounds (24). Promoting a process-based approach to culturally-sensitive psychotherapy may be key to facilitating a health equity model.

### How

Until recently, mental healthcare was typically delivered face-to-face and at a time and place that were most suitable for the provider. These delivery formats pose particular challenges to people from lower social classes and ethnic minorities (25), and perinatal BIPOC women are no exception. An emphasis on digital platforms to increase access to care is common, was raised by USPSTF, and has arguably revolutionized mental healthcare over the past 2 years of the pandemic.

The COVID-19 crisis has highlighted the essential and revolutionized role of telehealth and digital tools to offer direct-to-patient care that minimizes barriers (26). Evolving evidence that has grown over the pandemic due to widespread adoption of telepsychiatry, has shown that delivery of the same treatment through a digital platform can be as effective as in-person treatment, but preferred by patients and with more durable outcomes (27). Telemedicine and web-based interventions may offer a promising alternative for perinatal patients in terms of flexibility, efficiency, and cost, potentially increasing the accessibility and scalability of treatment (28). Remote psychotherapy can also be offered in patient-centered settings and schedules (i.e., at home and during weekends), while practical barriers (i.e., wait times and costs for parking or transportation) can be minimized, including for perinatal populations (29). There nonetheless remain questions that are critical to examine from a health equity lens. For instance, there may be some perinatal patients who may prefer or benefit more from in-person care because their home situation does not offer the needed privacy or safety to attend psychotherapy sessions. These potential barriers may be amplified for those who have multiple children, spouses working from home during the pandemic, or who are subject to intimate partner violence. Given the need for equitable care for perinatal populations, it is critical, ethical and timely to examine who may benefit more from in-person treatment compared to telemedicine.

### When

Although the USPSTF pointed out that the majority of effective psychotherapies were provided to women across the perinatal timeframe, a question remains as to whether the beneficial effects of delivering counseling to mothers during pregnancy are durable in the same women after delivery. This question takes on additional relevance since depressive symptoms that begin antenatally may worsen postpartum if left untreated (30). It has been demonstrated that when patients are screened serially, women who screen positive for depression during pregnancy are not the same women who subsequently screen positive after delivery. One interpretation is that antenatal interventions may have a lasting effect (31), such that effective interventions during pregnancy could impact postpartum depression, achieving a “multiplier effect” toward symptom resolution. Furthermore, there remain questions about considering perinatal mental health from a lifespan approach, including from a preconception conceptual framework. There is growing evidence to suggest that perinatal mental disorders are often preceded by mental health problems that begin before pregnancy, including in adolescence or young adulthood, and these maternal preconception disorders are associated with adverse effects for the mother, infant and child (32, 33). Likewise, equipping adolescents and youth—arguably the most vulnerable with long-term consequences (34)—with the needed tools may help facilitate a preventive approach to best cope with common symptoms of depression, anxiety and distress (35). A strong emphasis in this movement is self-care, including the application of evidence-based techniques such as relaxation and mindfulness, activity scheduling and structuring, and socially connecting to promote psychological wellbeing.

## Discussion

### Research Applications

Answers to these pragmatic questions may be on the horizon. Responsive to the call for creative solutions, relevant research is just now being undertaken to address the who, what, how, and when. Many of these elements of care form the aims of a recently funded Patient Centered Outcomes Research Institute study—a large-scale, non-inferiority randomized controlled trial^1^ (RCT) of both pregnant and postpartum women, comparing two delivery modes of a brief, evidence-based, behavioral activation intervention for depressive symptoms (telemedicine vs. in-person) provided by two different delivery agents (mental health specialists vs. trained non-mental health professionals). This study is exploring “what works for whom,” by examining who may benefit more from in-person psychotherapy compared to telemedicine. This multi-site, pragmatic trial is being implemented across real-world, primary care settings in Toronto, Canada; Chapel Hill, North Carolina; and Chicago, Illinois (36).

In addition, a National Institute of Mental Health (NIMH) funded trial is exploring the how and who with technology; in fact, this study's intervention is administered without direct involvement of any mental health professionals. This web-delivered product called MomMoodBooster (MMB)^2^, employs cognitive behavioral therapy-guided multimedia modeling and engaging activities and has already demonstrated efficacy in postpartum women (37). Using MMB adapted for delivery to the patients' smart phone, an RCT is being conducted to compare MMB to usual care during pregnancy and in the postpartum period, with specific content tailored to each timeframe. In essence, this is a sophisticated self-help program designed to enable depressed women to identify patterns in their thoughts in order to develop personal action plans that lead to helpful changes and reduced symptoms. This study responds to the “how” with technology and the “who,” by empowering patients themselves as the treating agents.

Another NIMH-funded RCT^3^ (35) is designed to examine the effectiveness of a group telehealth counseling intervention to reduce depressive symptoms among diverse ethnic groups of pregnant and postpartum women. Peer-supported group care has been determined to be effective more generally during pregnancy, especially among women with limited social and economic resources. Finally, a large trial focused on BIPOC women will be implemented across the USA to compare the effects of one psychotherapeutic intervention delivered through self-help, by a nurse, and treatment as usual (38). The study will include pregnant women and will examine the potential comparable effects of these groups to both prevent and potentially reduce perinatal depressive symptoms over time.

Examining these questions hold the promise for new models of care. If effective, these innovations could be highly scalable and incorporated into larger, stepped care systems to improve access to “personalized” counseling interventions.

### Clinical Applications

While we await the completion of the aforementioned trials, we must also implement existing evidence-based care more effectively and comprehensively at the regional level, as has been accomplished clinically in the UK (39). The Improving Access to Psychological Treatments (IAPT) program in the UK is a scalable model of evidence-based psychological treatments for depression and anxiety. Annually, over 1 million patients are provided for, 98% of whom are monitored in terms of a rigorous and automated monitoring system. Further, the program has demonstrated rigorous cost-effectiveness across the country (39). Within IAPT, there has been a focus on scaling up treatments for patients with perinatal depression. Specific efforts have demonstrated promising results for the effectiveness of partnerships to ultimately facilitate access to psychological interventions (40). Strategies included developing an integrated pathway from the antenatal clinic to IAPT, increasing public and healthcare professionals' awareness about the interventions, and providing training to Health Visitors (community public health nurses, registered midwives or nurses). What was most notable was the increase in self-referrals from perinatal women following partnerships, suggesting women were better able and empowered to access the needed support.

## Summary

As the COVID-19 pandemic highlights the gaps in our healthcare systems and transitions mental health care delivery into a virtual reality, ongoing research is needed to inform key users on what works among pregnant and postpartum women. More research is required to incorporate significant others, determine what works for whom, whether there are certain subgroups who benefit more from digital interventions and how they can be scaled from a health equity perspective. There is a clarion call to action to reduce the public health burden of perinatal depression throughout North America. We are encouraged by innovations for delivering psychological treatment to perinatal women and we are optimistic that scalable and long-term solutions may be just around the corner.

## Data Availability Statement

The original contributions presented in the study are included in the article/supplementary materials, further inquiries can be directed to the corresponding author/s.

## Author Contributions

DS drafted the initial version of this manuscript with significant contributions from SM-B, KS, and RS. All authors have critically reviewed this manuscript and provided consent for publication. The authors read and approved the final manuscript.

## Funding

This study was funded as a Pragmatic Clinical Study (PCS) by the Patient Centered Outcomes Research Institute (PCORI), PCS-2018C1-10621, and the National Institute for Mental Health (NIMH), 2R22 MH109191-02. DRS is also partially supported by an Academic Scholars Award from the Department of Psychiatry at the University of Toronto. The funders were not involved in the design of the study and is not involved in the collection, analysis, and interpretation of data, nor in writing the current manuscript.

## Conflict of Interest

The authors declare that the research was conducted in the absence of any commercial or financial relationships that could be construed as a potential conflict of interest.

## Publisher's Note

All claims expressed in this article are solely those of the authors and do not necessarily represent those of their affiliated organizations, or those of the publisher, the editors and the reviewers. Any product that may be evaluated in this article, or claim that may be made by its manufacturer, is not guaranteed or endorsed by the publisher.

## Footnotes

^1^ https://www.pcori.org/research-results/2018/scaling-psychological-treatments-perinatal-depression-and-anxiety-symptoms

^2^ https://projectreporter.nih.gov/project_info_description.cfm?aid=9622062&icde=43391628&ddparam=&ddvalue=&ddsub=&cr=2&csb=default&cs=ASC&pball=

^3^ https://projectreporter.nih.gov/project_info_description.cfm?aid=9660031&icde=43398087&ddparam=&ddvalue=&ddsub=&cr=1&csb=FY&cs=DESC&pball
